# AI-assisted heart failure management: A review of clinical applications, case studies, and future directions

**DOI:** 10.21542/gcsp.2025.6

**Published:** 2025-02-28

**Authors:** Abdullaah Idris-Agbabiaka, Muhammad Mehwar Anjum, Mehak Semy, Shree Rath, Muhammad Rizwan, Okam Onyinyechi Victoria, Amna Anwar, Folayemi Abiodun Iwaloye, Patrick Ashinze

**Affiliations:** 1 Department of Medicine, Texila American University, Guyana; 2 Sheikh Zayed Medical College Rahim Yar Khan, Pakistan; 3 Dr. D. Y. Patil School of Medicine, Nerul, Navi Mumbai, Maharashtra (400706), India; 4 All India Institute of Medical Sciences Bhubaneswar, India; 5 Babcock University Teaching Hospital, Nigeria; 6 Federal Medical College Islamabad, Pakistan; 7 Faculty of Clinical Sciences, College of Health Sciences, University of Ilorin, Ilorin, Nigeria

## Abstract

Heart failure is a major global health problem that affects over 64 million people and has significant economic costs. Early diagnosis and effective treatment are crucial, but traditional methods can be limited by the complexity and variability of symptoms. New approaches are needed to improve diagnosis and treatment, such as innovative biomarkers, advanced imaging, and personalized therapy. This study explores the application of artificial intelligence (AI) in heart failure diagnosis. The integration of AI in heart failure care holds transformative potential by enhancing diagnostic accuracy, predicting disease progression, and personalizing treatment plans through sophisticated algorithms and machine learning models. Technologies such as automated image analysis, natural language processing, and wearable devices enable continuous monitoring and timely interventions, improving patient outcomes and reducing hospital readmissions. Despite data privacy and algorithm transparency challenges, AI’s ability to process vast datasets and provide real-time insights represents a significant leap forward in heart failure management. This review emphasizes AI’s promising applications and future directions in reshaping heart failure care.

## Introduction

### Background of heart failure diagnosis and management

Heart failure is a complex disease that places a heavy burden on the healthcare system globally. It is one of the leading causes of hospitalization worldwide. As of 2017, an estimated 64.3 million people were affected by this condition^1^, and this number is expected to increase. Because of this, heart failure has been classified as a pandemic, imposing significant challenges on patients, their families, and healthcare services. The American Heart Association estimates that the cost of heart failure will increase from $20.9 billion in 2012 to $53.1 billion in 2030^2^.

### Importance of early detection and effective management

For HF, especially in the case of acute heart failure exacerbation, time is critical, as multiple studies have demonstrated increasing morbidity and mortality with late diagnosis, leading to delayed treatment^3,4^. However, establishing a diagnosis of heart failure is challenging. Heart failure is primarily a clinical diagnosis^5^, but the presentation of symptoms can be unspecific. For example, acute heart failure exacerbation patients may experience vague symptoms of dyspnea^6^. In elderly populations, diagnosis is further complicated by comorbidities and atypical symptoms^7^. With the new age of artificial intelligence (AI) and the integration of AI in healthcare, it is possible to use AI to help in the diagnostic process for heart failure.

### Overview of artificial intelligence in healthcare

Artificial intelligence represents a form of human-like intelligence exhibited by machines. Since its development in the 1950s^8^, AI has expanded into many fields, including healthcare. The application of AI in healthcare has been well researched and documented, especially in fields such as oncology and neurology. In 2019, Rodriguez-Ruiz et al. reported that an AI system could detect breast cancer on mammograms with an accuracy comparable to that of the average radiologist^9^. Research by ^10^ revealed that AI could identify people with Parkinson’s disease by analyzing their nocturnal breathing. AI was also used to assess disease progression and severity^10^.

The aim of this review is to provide a comprehensive exposition and evaluation of the current clinical applications, case studies, and future directions of AI-assisted heart failure management. It serves to compare AI-aided management with traditional methods, discuss the limitations, challenges and clinical prospects that belie AI-assisted treatment, elaborate the ethical implications and potential biases inherent in AI algorithms used for heart failure management and highlight the dearth of longitudinal studies that monitor the long-term impact of AI interventions on patient health and quality of life.

## Methodology

### Search strategy

We systematically searched the PubMed database for all articles published from January 2016 until May 2024. MeSH terms such as ‘artificial intelligence,’ ‘ai artificial intelligence,’ ‘machine learning,’ and ‘deep learning’ were combined via the Boolean operator OR. Finally, the MeSH term ‘heart failure’ was added to the search string using ‘AND’ to ensure the inclusion of articles employing AI technologies in patients with heart failure. Our search yielded 596 results, 71 of which were included in our study. Details of our search are given in Table 1.

**Table 1:** Search details and string used in the review.

Database	PubMed
Search Date	09th May, 2024
Search string	((”artificial intelligence”[MeSH Terms] OR ”machine learning”[MeSH Terms] OR ”deep learning”[MeSH Terms]) AND ”heart failure”[MeSH Terms]) AND (2016:2024[pdat])
Total results	596
Included articles	71

### Study selection

Predetermined inclusion and exclusion criteria were established before the review. Studies were considered eligible if they:

1. included original articles, including but not limited to original studies, cohort studies, case-control studies, cross-sectional studies, and randomized controlled trials. Included studies were critically analysed for methodological quality as obtainable for systematic reviews, meta-analyses and original studies;
2. included application of any AI model in prognostics, diagnostics, or treatment strategies for HF;
3. were written in the English language.

The exclusion criteria were gray literature, conference abstracts, editorials, commentaries, letters, and non-English articles.

## Artificial intelligence in heart failure diagnosis

### AI-based risk prediction models for heart failure

Artificial intelligence can be commonly used to help develop standardized predictive models that could help cardiologists improve patient-specific decision-making. Some of the widely used risk prediction models include:

#### Seattle heart failure model

This is the most commonly used risk prediction tool, developed via machine learning techniques to estimate the survival of patients with heart failure at 1 year, 2 years, and 3 years and the effects of adding medications or devices to the patient’s regimen^11^. The score reveals that NYHA class, ischemic etiology, diuretic dosage, ejection fraction (EF), systolic blood pressure (SBP), sodium levels, hemoglobin, percentage of lymphocytes, uric acid, and cholesterol all exhibited independent predictive capability. It facilitates prognosis estimation, increases the use of medications, and enhances compliance^11^. The SHFM effectively forecasts mortality risk in younger patients following hospitalization due to acute heart failure. However, patients aged 65 and older demonstrated a higher adjusted mortality risk for up to 18 months after discharge in comparison to those with ambulatory heart failure, reflecting the well-known post-hospital syndrome^11^.

#### Meta-Analysis Global Group in Chronic Heart Failure (MAGGIC) heart failure risk score

This model is not an AI-based model per se, but its score is used for risk stratification for both morbidity and mortality in heart failure patients with a preserved ejection fraction. It is clinically attractive because it includes routinely collected variables, is easy to use, and is effective at predicting 1-year and 3-year mortality in patients with heart failure^12^. A validation study noted that the addition of BNP or NT-proBNPto the MAGGIC risk score was beneficial in predicting more deaths in hospitalized patients with HF[13]. Another model, the European Society of Cardiology Heart Failure Long-Term Registry (ESC-HF-LT) risk score, works on similar principles^13^.

#### The Get with the Guidelines Heart Failure (GWTG-HF) risk score

The American Heart Association developed it to predict patient mortality in patients with acute heart failure. It provides prognostic prediction in the acute phase of hospitalization and during the chronic phase post-discharge^14^. While prognostic variables overlap in cardiac patients, regardless of the admission diagnosis, it also has proven to be of tangible reliability in prognosticating outcomes in patients with tricuspid regurgitation —and those who have undergone trans-catheter tricuspid valve repair^14^.

#### Acute Decompensated Heart Failure National Registry (ADHERE)

This AI-based risk prediction model, which happens to arguably be the world’s largest repository of acute heart failure datasets, was designed to bridge differences between knowledge and care by prospectively studying the characteristics, management, and outcomes of hospitalized patients with acute decompensated heart failure^15^. This study was intended to provide hospital teams with effective devices to improve the quality of care for these patients. Various parameters of these models are compared in Table 2.

**Table 2:** Variables used in SHFM, MAGGIC, and GWTF-HF.

MODELS			
Variable	SHFM	MAGGIC	GWTF-HF
Age	✓	✓	✓
Male Sex	✓	✓	
Race			✓
Body Mass Index	✓	✓	
Systolic Blood Pressure	✓	✓	✓
Heart Rate			✓
Hemoglobin	✓		
Total Cholesterol	✓		
Lymphocytes	✓		
Sodium	✓		✓
Serum Creatinine		✓	✓
BUN			✓
Uric Acid	✓		
Diabetes Mellitus		✓	
COPD		✓	✓
Ischemia (cause)	✓		
Current Smoker		✓	
NYHA Class	✓	✓	
Heart Failure duration <18 months		✓	
ACE inhibitor/ARB use	✓	✓	
Beta Blocker use	✓	✓	
Statin Use	✓		
Aldosterone Blocker use	✓		
Loop Diuretic Use	✓		
Allopurinol Use	✓		
Ejection Fraction	✓	✓	
Device Therapy	✓		
QRS duration	✓		

**Notes.**

Abbreviations SHFM Seattle Heart Failure Model MAGGIC Meta-Analysis Global Group in Chronic (MAGGIC) GWTF-HF The Get with The Guidelines-Heart Failure

### Automated image analysis for cardiac imaging

Several modalities have been developed and designed to assess cardiac structure and function. Some of these include:

#### Echocardiography

The most common, universally used technique to assess cardiac structure and identify those at greater risk of developing heart failure with previously diagnosed hypertension^16^ or slightly increased levels of natriuretic peptide (BNP: >50 pg/ml) (class IIa to IIb recommendation)^17^. Machine learning models such as recurrent neural networks (RNNs), mainly long short-term memory (LSTM) networks, can be used to detect a future diagnosis of heart failure in patients by evaluating sequences of echocardiogram frames with time to track function and structural changes in the heart^18^.

#### Automatic coronary artery calcium scoring

Coronary artery calcium is a direct marker of coronary atherosclerosis and an independent predictor of cardiovascular events and mortality. It can be evaluated via non-contrast CT^19^. The use of deep learning helps fully automate the tasks, leading to improved accuracy and efficiency.

#### Coronary computed tomography angiography (CCTA)

First line of diagnosis used to assess the severity of stenosis in patients with stable chest pain. Deep learning-based applications such as Hierarchical ConvLSTM have been developed and externally validated in a multicenter study of 1,611 patients (6,210 lesions) to accurately estimate the stenosis rate and reduce the inter-reader variability and interpretative error^20^.

#### Fractional flow reserve CT

Invasive fractional flow reserve (FFR) is used to assess coronary hemodynamics. Artificial intelligence in FFR-CT can replace tedious and time-consuming complex computer fluid dynamics (CFD) computations and reduce the time to analyze them^21^.

#### Cardiac magnetic resonance imaging for cardiac function

AI-based cardiac MR can perform rapid and automatic heart chamber segmentation and analyze heart function, thus providing fully automated, quality-controlled CMR analysis without supervision^22^.

### Natural language processing for extracting insights from clinical notes

NLP methods are powerful tools in healthcare systems and can potentially transform clinical decision support systems (CDSSs). They were developed to automatically alter clinical text into structured clinical data, which can then potentially guide healthcare providers in making clinical decisions, thus preventing or delaying disease onset. The goal of this method is to produce focused results that are specific to the problem^23^. Clinical natural language processing methods can be evaluated based on intrinsic criteria, that is, by directly measuring their performance in achieving their immediate objective, or on extrinsic criteria, where NLP then becomes a part of a more complicated process and its functionality in attaining the goal is checked^24^. It can facilitate data integration from clinical notes and other healthcare data resources, such as electronic healthcare records (HERs), laboratory tests, and radiology reports, and provide real-time support to healthcare providers in clinical decision-making, improving patient outcomes.

### Wearable devices and remote monitoring with AI integration

Wearable device technology allows healthcare providers to monitor disease dynamics remotely while simultaneously allowing them to intervene in a timely and efficient manner, potentially preventing hospitalizations secondary to adverse cardiac events and hemodynamic decompensation^25,26^.

The commonly used wearables in heart failure patients include pedometers and actigraphy devices, which collect data by analyzing patterns of physical activity. Healthcare providers and patients can then use this to modify lifestyle and therapy appropriately^27,28^.

Similarly, among device-based monitoring systems, the only FDA-approved remote monitoring system for patients with heart failure is the cardio-microelectromechanical system (CardioMEMS system; Abbott, Sylmar, USA). These implantable devices enable the monitoring of pulmonary arterial pressure through a sensory implant in the pulmonary artery^29,30^. It is mainly used in symptomatic patients with heart failure who have a history of hospitalization due to heart failure to reduce the risk of recurrent hospitalizations.

Other wearable devices assimilated with artificial intelligence include wearable seismocardiogram patches. These patches help proactively evaluate and manage heart failure patients^31^. They help identify heart failure patients’ conditions and treat them before they need hospitalization. Additionally, implantable devices such as implantable cardiac defibrillators, pacemakers, and cardiac pressure sensors can also be utilized to create algorithm-based risk prediction models that focus primarily on early diagnosis and treatment selection^32^.

## Artificial Intelligence in Heart Failure Management

### AI-based decision support systems for treatment planning

Heart failure (HF) is a multifaceted clinical syndrome and a disease that frequently results in insufficient care for patients and ineffective clinical tests. Its management is being changed by artificial intelligence (AI) through its ability to interpret and give significance to vast sets of data more quickly and efficiently; this helps to understand patterns of disease progression, classify its phenotypes, and observe outcomes with an enhanced degree of personalization^33^.

With the help of tools such as ML, DL, and neural networks^34^ incorporated with preexisting data, algorithms can be used to create models with the collaboration of patients and clinicians that include information on symptoms and lifestyle behaviors, blood test results, electrocardiogram recordings or ECGs, electronic health records, and cardiac imaging. Some of these models and algorithms, such as MARKER-HF, are discussed in a review by Blaziak et al. ^35^. Moreover, Chen et al. devised an AI-based heart failure treatment advisory system that may need more application and elaboration to prove its efficacy^36^.

### Personalized medication recommendations via machine learning

The application of machine learning for the personalized treatment and alleviation of heart failure can be delineated in four ways:

i. Optimizing pharmacological therapy: This can be accomplished by assessing the heterogeneity of the response to various heart failure medications. Ahmad et al. ^37^ reported that ML accurately predicted outcomes in a large dataset of HF patients, distinguishing four distinct phenotypes with varying responses to various medicines.

ii. Patient selection for devices: Cardiac resynchronization therapy (CRT) does not work for approximately one-third of patients with HF. An analysis revealed that ML algorithms could sort a diverse group of HFs into four apparent phenotypes, which might increase the response rate to CRT^38^. Similarly, implantable cardioverter defibrillators (ICDs) decrease the risk of sudden cardiac death in patients with HF. An ML-based phenomapping study demonstrated that it can recognize separate phenotype subgroups^39^, facilitating the application of personalized ICD devices for these patients.

iii. Closing the care gap and Clinical Trial recruitment: Jing et al., in their study, discuss the role of AI in predicting benefits in closing the care gap and how it could help set parameters and categorize patients to encourage or discourage recruitment into clinical trials^40^.

iv. Patient-centered care and rehabilitation: The advent of telemedicine has facilitated patient care^41^. AI can further guide the development of focused interventions and increase patient empowerment through shared decision-making and enhanced self-disease management efficacy. AI can improve organizational care by reducing patient waiting times and per capita costs while increasing accessibility, productivity, and overall patient experience^42^.

### Predictive analytics for hospital readmission and disease progression

Shah et al., Gevaert et al., and Bose et al. ^43–45^ improved the existing classification system of HF by clustering study participants via ML into phenotypes according to various parameters, such as ECGs, biodata, etc., and outcomes. This helps researchers study and better understand disease progression, predict disease outcomes, and predict patient prognosis. Risk stratification is crucial for HF, and ML can be especially useful in forecasting clinical results. Research by Golas et al. and Kwon et al. ^46,47^ demonstrated that a DL-based algorithm performed better than traditional methods. It predicted the number of readmissions within 30 days. It estimates other mortality parameters more precisely than existing scores, such as the GWTG or MAGGIC score.

## Comparison with traditional heart failure management

### Comparison with manual risk assessment tools

Manual risk assessment tools have been used as the mainstay of heart failure management^48^. The approach uses regression models to predict the occurrence of adverse events and establish the relationships between specific variables and the disease course^49^. These manual risk assessment tools have helped identify and characterize the impact of demographic features, comorbidities, and biomarkers on the pathogenesis and progression of heart failure^50^. However, while they have been valuable in assessing the risk associated with heart failure, these techniques have their limitations, as they rely on a limited amount of data for analysis, and their application in population-based studies involving a vast number of data variables is limited^50^. On the other hand, AI-driven models leverage advanced learning machine techniques to analyze large datasets^51^.

### Comparison with conventional treatment protocols

An AI-driven model analyses vast amounts of population-based data obtained from other people as well as real-time data, particularly from the patients themselves^52^. Additionally, the system’s adaptability allows room for improvement and changes as the patient’s condition either improves or worsens^53^.

### Comparative advantages of AI in real-time monitoring and decision-making

Traditional approaches to monitoring and decision-making in heart failure management depend significantly on periodic clinical visits and manual assessment of patient data^50^. In contrast, however, most AI-driven solutions allow patients to use devices that act as sensors, collect real-time data, and incorporate them into AI algorithms^54^, allowing clinicians to intervene before adverse events occur^55^.

## Clinical Applications and Case Studies

### AI-assisted heart failure management in clinical settings

A study in 2019 revealed that a deep neural network model outperformed cardiologists in diagnosing electrocardiogram (ECG) abnormalities, indicating that AI has the potential to make accurate diagnoses^56^.

Similarly, large language models can help patients generate individualized healthcare recommendations that explain the patient’s disease, treatment options, risks, and benefits^57^. Deep language models have been noted in real life clinical settings in accurately prognosticating treatment outcomes using the MAGGIC score^33^.

Early diagnosis of heart failure can help prevent adverse outcomes. A study revealed that AI has proven useful in classifying heart failure phenotypes^33^, and has interpreted chest X-rays to aid in the early diagnosis of HF, which may be challenging at an early stage because of nonspecific symptoms, particularly HF with preserved ejection fraction^58^.

### Remote monitoring and telemedicine with AI integration

In Italy^59^, connected care is gaining momentum in digital healthcare, putting patients at the system’s center. The connected care operating model is an excellent example of which all central and regional institutions converge. It enables the sharing of patient information among all the staff involved in the treatment process. Electronic health records are among the prime examples of digitizing and integrating technology into healthcare^60^. A randomized clinical trial^29^ involving 716 patients with a mean age of 65 years and a mean ejection fraction of 26% was performed. The primary outcome measure was a composite clinical score combining all-cause death, overnight hospital admission for heart failure, NYHA class change, and patient global self-assessment. This study suggests that telemonitoring is feasible and should be used in clinical practice.

### Case studies demonstrating improved patient outcomes

In 2019, Hannun et al.’s study^56^ revealed that deep neural network models outperformed cardiologists in diagnosing ECG abnormalities and arrhythmia. The results suggest that AI and deep learning can improve ECG analysis efficiency and accuracy and support expert-human interpretation, reducing the number of incorrect diagnoses and prioritizing urgent cases.

A case study discussed a large neural network that uses ECG and echocardiogram data from patients to detect ventricular dysfunction (ejection fraction [EF] ≤ 35%) based on ECG readings. Over a median follow-up period of 3.4 years, patients flagged by the AI-ECG as having issues, but who had normal echocardiograms (false positives), were four times more likely to develop left ventricular dysfunction compared to those who were confirmed to have normal EF by both the AI-ECG and echocardiography (true negatives)^60^. This indicates that the network can not only detect existing left ventricular dysfunction but also identify abnormal ECGs before the condition develops.

In another study, researchers used AI and convolutional neural networks to analyze wearable ECG monitors to predict atrial fibrillation. The AI-enabled ECG showed an accuracy of 79.4%, indicating that it could identify at-risk individuals with atrial fibrillation during normal sinus rhythms^61^.

## Advantages and limitations of AI in heart failure care

### Improved diagnostic accuracy and early intervention

The vast majority of people with heart failure are diagnosed only following emergency hospital admission, underscoring the challenge posed by this disease to many clinicians^62^. However, studies have shown that when used alone to diagnose heart failure, AI-generated results have high concordance rates with the actual conditions of the patients studied^63^. When clinicians incorporated AI tools in the diagnosis of heart failure, they observed nearly 100% accuracy^64^.

#### Potential for cost savings and resource optimization

Another compelling advantage of AI in heart failure care is its potential for cost savings and resource optimization^63^. The use of AI in the diagnosis of this condition significantly streamlines diagnostic processes, aiding the development of personalized treatment plans^65^ and allowing healthcare providers to allocate resources more effectively^66^. For example, AI-driven support systems can assist clinicians in individualizing treatment regimens for the patient in question, optimizing medication usage, and minimizing unnecessary procedures.

### Challenges in data privacy, bias, and algorithm interpretability

The use of AI has several drawbacks. Its novelty may explain some drawbacks of AI use in heart failure care^67^. Because the training of AI algorithms requires that patients’ data be fed to the AI, issues regarding the safety and confidence of these data are a significant challenge faced by the system^68^. Additionally, biases arising from these training data or the algorithm design itself may result in disparities in diagnosis, treatment recommendations, or patient outcomes across demographic groups^68^. Furthermore, because many AI models operate in what may be described as a “black box,” clinicians may find it challenging to understand the rationale behind their recommendations, thus affecting their interpretability^66^.

## Future directions and research opportunities

### Advancements in AI technology for heart failure care

ML and DL technologies are the heart of AI and are being developed on a massive scale for further implementation in healthcare. Particularly for heart failure, these technologies have already been used to aid in the diagnosis and management of HF, as delineated in the review thus far, and newer studies and models are still being developed:

i. A novel machine learning algorithm combining photoplethysmography and ECG is being developed to detect and diagnose heart failure. The model showed a precision of 97.2%, thus demonstrating the system’s high performance^69^.

ii. Another model developed by Dhingra L et al. used ECG data to predict risk in patients with heart failure and achieved excellent results^58^. AI can also harness electrical signals generated from ECGs and bioimpedance to monitor patients in a decompensated state^70^.

iii. Decision engines have also been built to provide doctors with recommendations for treatment courses for patients with HF. All the recommendations provided by the system were in line with internationally accepted guidelines and matched with doctors’ recommendations in approximately 94% of the cases^59^.

### Integration of AI with electronic health records and wearable devices

An AI model fitted to wearable devices could help detect left ventricular systolic dysfunction^71^. Another device tested in a UK cohort of patients predicted hospitalization and death in patients with heart failure^58^. Furthermore, a wearable AI device was able to detect hospitalization, with a sensitivity of nearly 80% and specificity of 85%. Patients were readmitted within a week of the alert given by the device, showing the device’s accuracy and early detection rate^60^. This device also offers a scope for remote monitoring, allowing active response and guidance during emergencies. More research and investment of resources into such devices would facilitate a smoother transition into telemedicine and a healthcare structure better corroborated with AI.

### Promising areas for further investigation in AI-driven heart failure management

Hybrid deep learning systems are a novel field of technology that combines multiple deep learning models^61^. These devices can help preprocess data before it fits into deep learning algorithms for efficient and better prediction results. Devices are also being actively developed, and further development must be undertaken to improve their specificity and sensitivity.

### Cost-effectiveness of AI in healthcare settings

A systematic analysis revealed that current impact assessments of AI technology in healthcare have significant methodological shortcomings. These deficiencies suggest that the evaluations conducted thus far may not provide a complete or accurate picture of the economic implications of using AI in medical settings^72^. To make informed economic decisions about whether to adopt or reject AI technologies in healthcare, future evaluations must incorporate more thorough and detailed economic analyses. This means going beyond basic assessments to include a wider range of factors, such as costs, benefits, long-term impacts, and potential risks. By doing so, stakeholders will be better equipped to understand the financial and operational consequences of implementing AI technologies in healthcare, ultimately leading to more informed decision-making.

## Limitations of study

### Methodological selectivity

The studies reviewed may vary widely in terms of design, sample size, and methodological rigor. Although 71 articles were included, the selection criteria may introduce biases. For instance, studies with smaller sample sizes or less robust methodologies could skew the effectiveness of AI applications reported in this review. The reliance on published literature may also lead to publication bias, where positive results are favored in the literature, potentially overlooking negative or inconclusive findings.

### Resource constraint for a more elaborate analysis of existing variables

The quality of the data used in AI training and evaluation is paramount. Many studies may base their models on homogeneous populations or datasets that lack diversity regarding demographics such as age, ethnicity, and comorbidities. Consequently, the applicability of AI findings across varied patient populations in real-world clinical settings remains uncertain. Limited diversity can lead to algorithmic biases, where AI models may not perform equally well across different demographic groups, potentially exacerbating health disparities. The transition from traditional heart failure management to AI-assisted approaches poses significant integration challenges within the existing healthcare infrastructure. Many institutions may lack the necessary resources, technological support, or training to implement AI-based tools effectively. Additionally, the integration of AI systems with electronic health records and existing clinical workflows is often complex, potentially limiting the practical utility of these technologies in routine clinical practice.

### Ethico-legal considerations

The review highlights ethical implications surrounding the use of AI in healthcare, yet it may not delve deeply into the full breadth of ethical, legal, and regulatory challenges. Issues such as patient consent for data usage, ownership of AI-derived insights, and liability in the event of AI misdiagnosis or mismanagement remain largely unaddressed. These challenges can hinder the acceptance and adoption of AI technologies among healthcare providers and patients alike.

### Limited longitudinal studies

As noted in the overview, there is a marked deficiency of longitudinal studies that monitor the long-term impact of AI interventions on patient health and quality of life. Most current evaluations appear to focus on short-term outcomes, potentially overlooking long-term effects, deterioration of health, or the sustainability of AI-based treatment strategies. By not examining these long-term outcomes, the review does not provide a complete understanding of the effectiveness and safety of AI applications in HF management.

### Limited focus on economic evaluation

While our paper touches upon the economic implications of AI integration, it lacks a thorough economic analysis that encompasses overall cost-effectiveness within various healthcare settings. Future evaluations and studies need to incorporate detailed economic assessments of AI technologies, taking into account both direct costs (such as implementation and maintenance) and indirect costs (like long-term patient outcomes and resource allocation).

## Conclusion

The applications of artificial intelligence in heart failure initial workups are expanding rapidly. It can potentially aid in the early diagnosis of heart failure and the classification of phenotypes. It also has the potential to help clinicians with ongoing management, including medication titration, device therapy, optimized management, clinical trial enrollment, and prognosis estimation.

As highlighted in our review, implementing AI in future healthcare models has several advantages. It improves the timely diagnosis of heart failure, significantly improving patient prognosis and survival rates. The use of AI also has the potential to tailor management plans according to the patient’s clinical picture.

However, challenges, such as confidentiality and ethics, are involved in the use of patient data to develop algorithms; hence, there is a new need to develop security policies. Another issue is the new requirement for clinicians to be trained to interpret and understand the rationale of these algorithms’ basis while using them to revolutionize HF management. These issues may currently delay the deployment of AI in healthcare.

Further work on interpretability is needed before it can be incorporated into medicine successfully. Possible avenues to explore include the use of ECG data to predict the possibility of HF and cardiac imaging analysis via deep learning for a confirmed diagnosis of HF, which could aid clinicians and improve early detection rates of HF. However, implementation strategies remain in the early stages of development, as more background knowledge needs to be available to increase clinicians’ confidence in using AI to aid in the diagnosis and management of HF patients. As this remains uncertain, more research is needed in the future on the added value that AI provides to clinicians in their work. We also need to establish a structured implementation process in the future for healthcare institutions to help move forward.

## Declarations

### Ethics approval and consent to participate

Not applicable

### Consent for publication

Not applicable

### Availability of data and material

Not applicable

### Funding

Not applicable

### Authors’ contributions

Conceptualisation, Writing of Initial and Final Draft, Initial Review: A.I-A

Writing, Editing, Data Collation: All authors

Final review, Validation and Supervision: P.A

## Competing Interests

We declare NO competing interests

## Acknowledgement

The authors would like to acknowledge THE LIND LEAGUE, Nigeria for providing the invaluable resources to kick start, culminate and leverage this research project while also enabling our capacities.
